# Optic disc microvasculature dropout in primary open-angle glaucoma measured with optical coherence tomography angiography

**DOI:** 10.1371/journal.pone.0201729

**Published:** 2018-08-07

**Authors:** Tadamichi Akagi, Linda M. Zangwill, Takuhei Shoji, Min Hee Suh, Luke J. Saunders, Adeleh Yarmohammadi, Patricia Isabel C. Manalastas, Rafaella C. Penteado, Robert N. Weinreb

**Affiliations:** 1 Hamilton Glaucoma Center, Shiley Eye Institute, Department of Ophthalmology, University of California San Diego, La Jolla, CA, United States of America; 2 Department of Ophthalmology and Visual Sciences, Kyoto University Graduate School of Medicine, Kyoto, Japan; 3 Department of Ophthalmology, Saitama Medical University, Saitama, Japan; 4 Department of Ophthalmology, Haeundae Paik Hospital, Inje University, Busan, South Korea; Bascom Palmer Eye Institute, UNITED STATES

## Abstract

**Purpose:**

To evaluate microvasculature dropout in the optic disc (Mvd-D) using optical coherence tomography angiography (OCTA) and investigate factors associated with Mvd-D in primary open-angle glaucoma (POAG) eyes.

**Methods:**

One hundred twenty-three eyes of 123 POAG patients were included from the Diagnostic Innovations in Glaucoma Study. The 3.0×3.0-mm optic nerve head OCTA scans were acquired using a spectral-domain OCT instrument. Images with whole-signal-mode were evaluated. Eyes were classified into 3 categories (Mvd-D, pseudo-Mvd-D, and no Mvd-D). Mvd-D and pseudo-Mvd-D had complete loss of OCTA signals on the temporal side of the optic disc on the en face projection image. They were distinguished base on the visualization of the anterior lamina cribrosa in the horizontal B-scans of that area. No Mvd-D was defined when no complete signal loss of OCTA signals was observed. Covariates including focal lamina cribrosa defects assessed by swept-source OCT and microvasculature dropout in the parapapillary region (Mvd-P) were analyzed.

**Results:**

Forty-two, 37, and 44 eyes were identified as having Mvd-D, pseudo-Mvd-D, and no Mvd-D, respectively. The eyes with Mvd-D showed significantly lower intraocular pressure, worse visual field mean deviation, larger cup-to-disc ratio, thinner circumpapillary retinal nerve fiber layer (cpRNFL), and lower circumpapillary vessel density within the RNFL than the eyes with pseudo-Mvd-D or the eyes without Mvd-D. Multivariable logistic regression analysis showed significant associations of Mvd-D with larger cup-to-disc ratio (OR, 1.08; *P* = 0.001), worse visual field mean deviation (OR, 1.09; *P* = 0.048), higher prevalence of focal lamina cribrosa defect (OR, 9.05; *P* = 0.002), and higher prevalence of Mvd-P (OR, 10.33; *P* <0.001).

**Conclusions:**

OCTA-derived Mvd-D was strongly associated with the presences of Mvd-P and focal lamina cribrosa defects, and these 3 findings were topographically associated with each other.

## Introduction

Ocular blood flow is thought to have an important role in the pathology of glaucoma.[1–3] In particular, vascular dysfunction in the optic nerve head (ONH) has been proposed as a contributing factor to the development and progression of glaucoma.[3] With fluorescein angiography, filling defects in the ONH have been reported in eyes with glaucomatous neuropathy.[4–8] However, fluorescein angiography is difficult to perform in a clinical setting as it is invasive and time consuming. Alternative imaging methods have been widely investigated, but it has been a challenge to identify clinically feasible ones until recently with the advent of optical coherence tomography angiography (OCTA).

Imaging of the perfused microvasculature of the optic disc with OCTA provides qualitative and quantitative information that previously has not been possible to acquire. Moreover, in addition to being non-invasive, the actual measurements are performed relatively quickly.[9–12] Peripapillary vessel density using OCTA has been shown to be reduced in glaucoma eyes and has been proposed as a diagnostic tool for glaucoma.[13,14] Further, it recently has been reported that dropout of the deep-layer microvasculature within areas of parapapillary atrophy is associated with systemic and ocular factors including focal lamina cribrosa (LC) defects in primary open-angle glaucoma (POAG) patients.[15] Formation of a focal LC defect is thought to be derived from structural changes at and around the LC and might be the cause of mechanical rupture of small blood vessels.[16–23] It was suggested that the significant association between microvasculature dropout (Mvd) in the parapapillary deep-layer (Mvd-P) and focal LC defects is caused by disruption of the laminar beams during the development of focal LC defects because both microvasculature in the parapapillary region and LC are downstream from the short posterior ciliary artery.[23] Considering these anatomic relationships, it is not surprising that focal LC defects affect microvasculature structures in the parapapillary area, as well as within the optic disc. However, there is little information regarding the relationship among Mvd within the optic disc (Mvd-D), Mvd-P, and focal LC defects.

The purpose of the current study was to evaluate and characterize the Mvd-D in POAG patients using OCTA, and also to clarify the relationship between Mvd-D and clinical features including focal LC damage and Mvd-P.

## Materials and methods

### Study subjects

POAG patients from the Diagnostic Innovations in Glaucoma Study (DIGS) (ClinicalTrials.gov identifier, NCT00221897) who underwent OCTA (Avanti with the Angiovue; Optovue Inc., Fremont, CA), spectral-domain OCT (SD-OCT) ONH imaging (Avanti; Optovue Inc.), and swept-source OCT (SS-OCT) (DRI OCT-1; Topcon, Tokyo, Japan) were included in this observational cross-sectional study. The included patients completed OCTA examinations from January, 2015 to September, 2016. Details of the DIGS protocol have been described previously.[24] This study was approved by the Institutional Review Board at the University of California, San Diego, and conformed to the tenets of the Declaration of Helsinki and the Health Insurance Portability and Accountability Act. Informed consent was obtained from all participants.

All participants underwent an ophthalmologic examination, including assessment of best-corrected visual acuity, refractive error, slit-lamp biomicroscopy, intraocular pressure (IOP) measurement with Goldmann applanation tonometry, gonioscopy, central corneal thickness (CCT) measured with ultrasound pachymetry (DGH Technology, Inc., Exton, PA), axial length measured by the IOLMaster (Carl Zeiss Meditec, Dublin, CA), dilated fundus examination, simultaneous stereophotography of the optic disc, standard automated perimetry (Humphrey Field Analyzer; 24–2 Swedish interactive thresholding algorithm [SITA]; Carl-Zeiss Meditec), SD-OCT, OCTA, and SS-OCT. The IOP obtained on the day of the OCTA examination was used in this analysis.

POAG was defined as the presence of glaucomatous optic nerve damage (i.e., the presence of focal thinning, notching, or localized or diffuse atrophy of the retinal nerve fiber layer [RNFL]) and associated repeatable visual field (VF) damage. Glaucomatous VF damage was defined as a Glaucoma Hemifield Test outside normal limits or a pattern standard deviation (PSD) outside 95% normal limits confirmed on 2 consecutive, reliable (fixation losses and false-negatives ≤33% and ≥15% false-positives) tests.

Eyes with a history of intraocular surgery (except for glaucoma surgery or uncomplicated cataract surgery), secondary causes of glaucoma, nonglaucomatous optic neuropathies, vascular or nonvascular retinopathies, and other ocular or systemic diseases known to impair the VF were excluded from the investigation. When both eyes were eligible, one eye from each patient was randomly selected for analysis.

Two blood pressure (BP) measurements obtained in a resting, seated position were taken at least 5 minutes apart using an Omron Automatic (model BP791IT; Omron Healthcare, Inc., Lake Forest, IL) instrument. Mean ocular perfusion pressure (MOPP) was calculated as MOPP = 2/3 (1/3 systolic BP + 2/3 diastolic BP)—IOP.

### Standard automated perimetry

All participants underwent VF testing using the 24–2 pattern SITA on the Humphrey Field Analyzer (Carl Zeiss Meditec) within 6 months of imaging. Only reliable tests (33% fixation losses and false negatives, and 15% false-positives) were included. The quality of VF tests was also reviewed by the Visual Field Assessment Center staff to identify and exclude VFs with evidence of inattention or inappropriate fixation, artifacts such as eyelid and lens rim artifacts, fatigue effects, and abnormal results caused by diseases other than glaucoma.[25]

### SD–OCT imaging

The Avanti SD-OCT system has a light source with a 70-kHz axial line rate and a center wavelength of 840 nm. The axial and transverse resolutions of this system were approximately 5 μm and 15μm. The acquired optic disc cubes consisted of 304 B-scans containing 304 A-scans each. The ONH map consists of a 6.0×6.0-mm cube scan which is used to automatically calculate disc area and cup-to-disc (C/D) ratio. In addition, average circumpapillary RNFL (cpRNFL) thickness is calculated in a 10-pixel-wide band along a circle of 3.45 mm in diameter centered on the ONH. Only good-quality images with a signal strength index (SSI) of 37 or more and without segmentation failures or artifacts were included.[13–15]

### OCTA imaging

The OCTA images were acquired using the Avanti SD-OCT system and analyzed with the Angiovue software. Each of the acquired optic disc cubes (3.0×3.0-mm and 4.5×4.5-mm) consisted of 304 clusters of 2 repeat B-scans containing 304 A-scans each. A split-spectrum amplitude-decorrelation angiography algorithm was employed to improve the signal-to noise ratio by splitting the spectrum to generate multiple repeat OCT frames from the 2 original repeat OCT frames.

Image quality of all scans was reviewed according to a standard protocol established by the Imaging Data Evaluation and Analysis reading center at the Hamilton Glaucoma Center.[24] Trained graders reviewed scans and those with poor image quality, as defined by the following criteria, were excluded: (1) SSI <48 (1 = minimum, 100 = maximum), (2) poor clarity, (3) residual motion artifacts visible as an irregular vessel pattern or disc boundary on the en face angiogram, (4) a local weak signal, and (5) RNFL segmentation errors.

### Vessel density measurements using OCTA

Vessel density within the peripapillary RNFL in scans with a 4.5×4.5-mm field of view centered on the ONH was used to measure circumpapillary vessel density (cpVD) as previously reported.[13,14] Average cpVD was automatically calculated in the RNFL in a region defined as a 750-μm-wide elliptical annulus extending from the optic disc boundary using AngioVue software.

### Microvasculature dropout in the optic disc and parapapillary region

**Fig 1:**
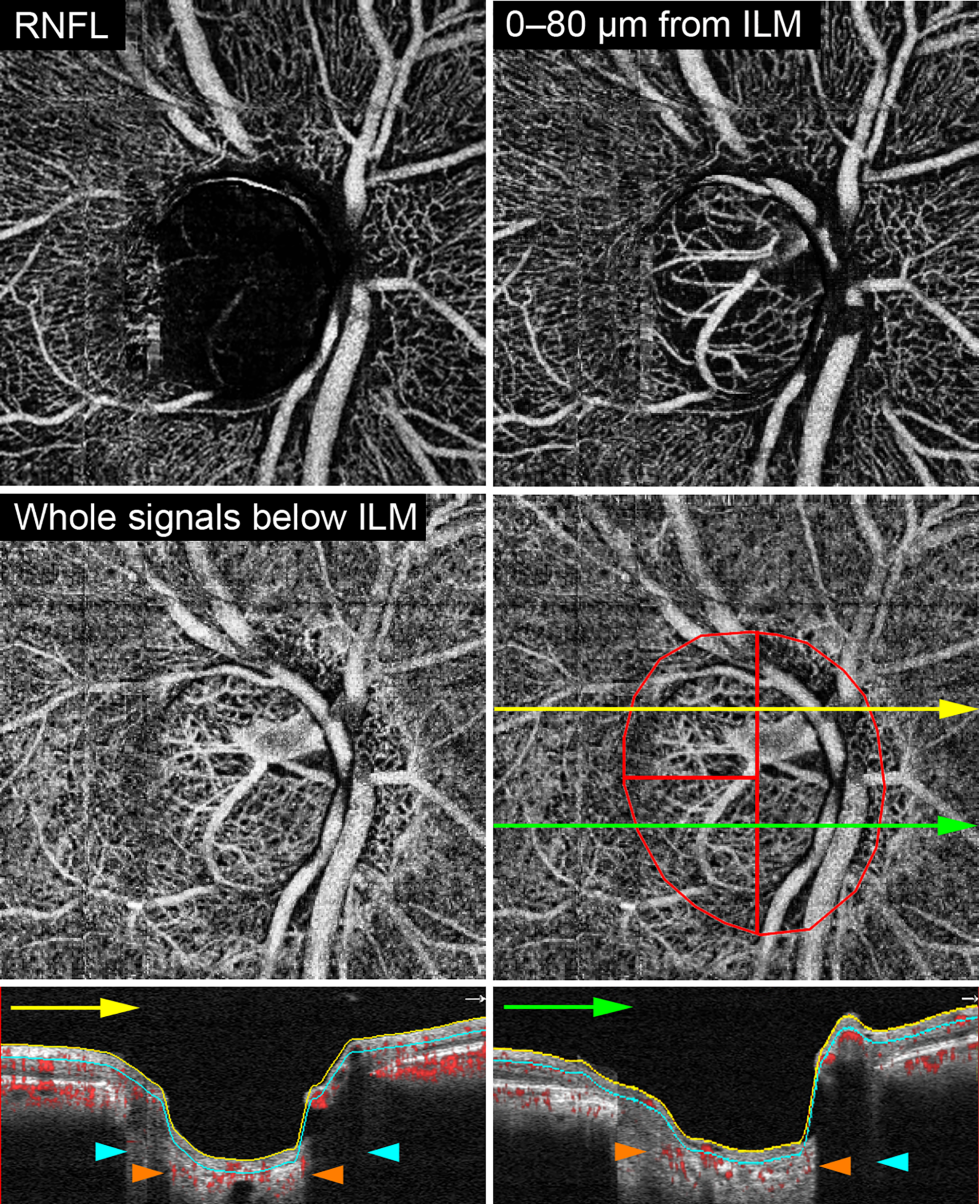
Optical coherence tomography angiography (OCTA) microvasculature images of optic nerve head in glaucoma eye without microvasculature dropout inside the optic disc. OCTA en face projection images in the retinal nerve fiber layer (RNFL) **(Top left)**, superficial layer **(Top right)**, and the whole-depth **(Second row)**. The red ellipse indicates the optic disc boundary and red lines show the boundaries of evaluation areas. Superotemporal and inferotemporal regions were used for evaluation of microvasculature dropout. The yellow and green lines indicate the location of the B-scans in the bottom row. **Bottom,** Cross-sectional angiogram images overlying the B-scan images. Microvasculature in the anterior portion of lamina cribrosa is visualized in the area shown by orange arrowheads, while the anterior lamina cribrosa and microvasculature cannot be detected because of vessel shadowing in the area shown by aqua arrowheads. Yellow and aqua borders show the inner limiting membrane (ILM) and 80 μm below the ILM, respectively.

The Mvd-D and Mvd-P were evaluated on the 3.0×3.0-mm ONH en face projection image using the OCTA whole-signal mode image, which was constructed from all of the OCTA signals below the inner limiting membrane (ILM) as shown in Fig 1.[26] The optic disc boundary was defined manually as the inner margin of the peripapillary scleral ring identified on scanning laser ophthalmoscopy (SLO) images acquired at the same positions as the OCTA.[27,28] The centroid of the optic disc was determined by using ImageJ software (National Institutes of Health, Bethesda, MD) and only the temporal side of the vertical line passing through the centroid of the optic disc was used for the analysis because the visualization of deep structures using OCT has been limited at the nasal side of the optic disc.[21,29,30] The horizontal line passing through the centroid of the optic disc was used to determine the location (superior or inferior) of the Mvd-D and Mvd-P.

**Fig 2:**
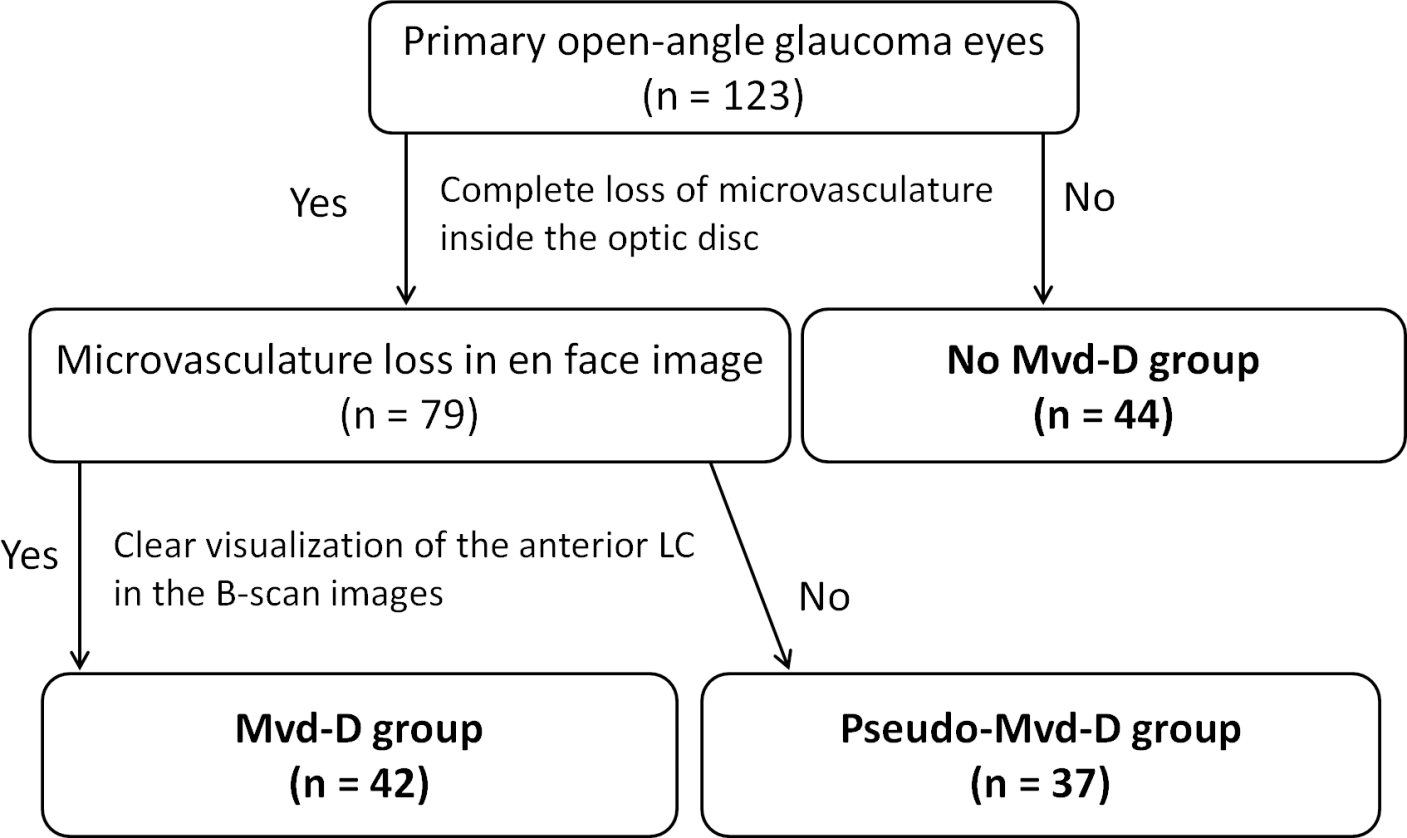
Flowchart determining the classification based on microvasculature dropout inside the optic disc (Mvd-D).

To classify the eyes into 3 groups based on Mvd-D, first the complete loss of OCTA signals, which was 200 μm or more wide and 100 μm or more in length, was evaluated on the temporal side of the optic disc on the en face projection image for all included eyes (Figs 1 and 2). If there was complete loss of OCTA signals, the eyes were further evaluated to determine whether they should be classified as Mvd-D group or pseudo-Mvd-D group; otherwise, the eyes were classified into no Mvd-D group (Fig 2). To decide whether eyes should be assigned to the Mvd-D group or pseudo-Mvd-D group, the visualization of the anterior LC was checked in the horizontal B-scan images at the area with complete loss of OCTA signal in all Mvd-suspect eyes. When the anterior LC portion was clearly visualized in the horizontal B-scan images of that area, the eyes were classified as Mvd-D. If the anterior LC portion was not clearly visualized, the eyes were assigned to the pseudo-Mvd-D group and the reason for poor visibility of the anterior LC was recorded. To determine clear visualization for Mvd-D, the anterior LC portion in the area with complete loss of OCTA signal was required to be clearly visualized in at least 100 μm in length (6 consecutive horizontal B-scans) and also to be 200 μm or more long in at least 1 scan. In eyes of Mvd-D group, there should be microvascular dropout in the anterior LC portion as well as the prelaminar tissue in the area.

Mvd-P was defined as a complete loss of the choriocapillaris or the microvasculature within the scleral flange on both 3.0×3.0-mm ONH en face and horizontal vessel density maps as previously reported.[15,23] Briefly, dropout was required to be present in at least 100 μm in length (6 consecutive horizontal B-scans) and also to be 200μm or more in diameter in at least 1 scan. The reason why the numbers of required consecutive horizontal B-scans were different between this study and the previous report[15,23] was the difference in OCTA scanning area (this report, 3.0×3.0-mm; the previous report, 4.5×4.5-mm).

In summary of the definition of no Mvd-D, pseudo-Mvd-D, and Mvd-D

1. No Mvd-D: No complete signal loss of OCTA signals is observed on the temporal side of the optic disc on the en face projection image.

2. Pseudo-Mvd-D: Complete loss of OCTA signals is observed on the temporal side of the optic disc on the en face projection image, but the anterior LC portion is not well visualized in that area.

3. Mvd-D: Complete loss of OCTA signals is observed on the temporal side of the optic disc on the en face projection image, and the anterior LC portion is well visualized in that area.

**Fig 3:**
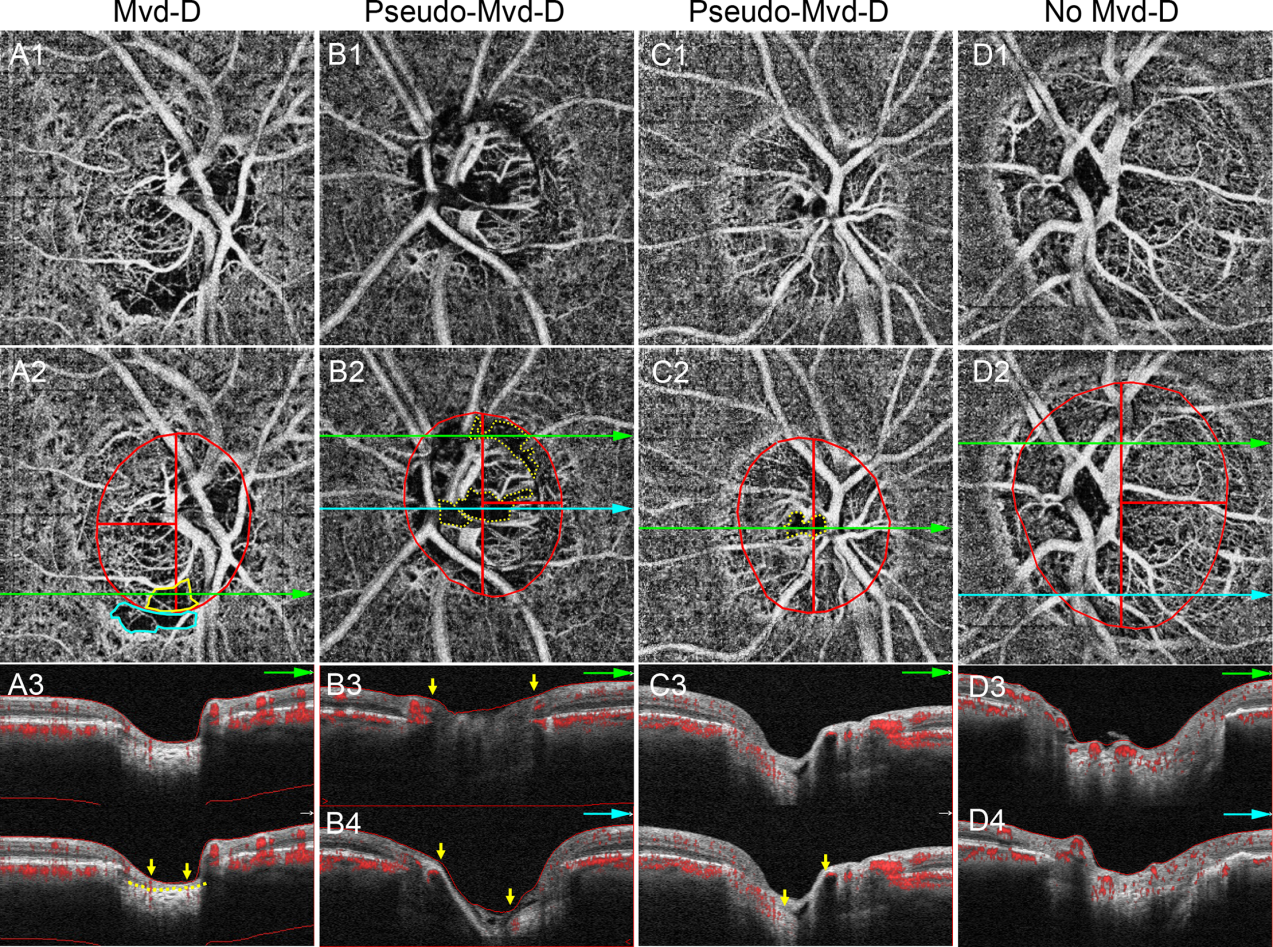
Examples of eyes with microvasculature dropout (Mvd) inside the optic disc (Mvd-D), pseudo-Mvd-D, and no Mvd-D. **A**, The Mvd-D and Mvd in the parapapillary region (Mvd-P) are enclosed by yellow and aqua border, respectively. **A3**, Anterior lamina cribrosa (LC) surface is well visualized as shown by yellow dotted line. Yellow arrows indicate the borders of the Mvd-D. It should be noted that the area between 2 yellow arrows does not contain any microvasculature signal in the prelaminar tissue and anterior portion of LC. **B,C**, Pseudo-Mvd-Ds are shown by yellow dotted border. Anterior LC could not be observed between 2 yellow arrows, which indicate the borders of the pseudo-Mvd-D, because of shadowing by neuroretinal rim (**B3**) and large retinal vessel (**B4, C3**). **D**, No Mvd-D is observed in the temporal side of the optic disc. Optic disc border is shown by red ellipse and the border for evaluation is shown by red lines (**A2―D2**).

Detection of Mvd-D and Mvd-P was performed by 2 independent observers (T.A., T.S.) masked to the patients’ baseline characteristics (Fig 3). Discrepancies between the two observers were resolved by consensus; if consensus could not be reached, the participant was excluded from the analysis.

### SS-OCT imaging

Images centered on the optic disc were obtained over a 6×6-mm area with DRI OCT-1 to determine the presence of focal LC defects. Details on the device have been described elsewhere.[16,21] Briefly, this SS-OCT system used an axial scan rate of 100,000 Hz with a wavelength-sweeping laser with an approximately 100-nm tuning range centered at 1050 nm, yielding 8 μm axial resolution in tissue. Transverse resolution was set to approximately 20 μm. Three-dimensional raster scan consisted of 256×256 A-scans. Poor-quality images with clipped or poorly focused scans, motion artifacts, quality score of less than 50, poorly visible LC, or with segmentation failure of the choroidal layer were excluded.[31,32] Poor visibility of the LC was defined as less than 70% visibility of the anterior laminar surface within the Bruch’s membrane opening.[15,23,32]

In determining the presence of focal LC defects, 2 independent observers (T.A., T.S.), masked to the clinical information of the study participants, reviewed the horizontal B-scan and en face SS-OCT images. The evaluation of LC defects was performed as previously described.[15,17–20,23] Focal LC defects were required to be 100 μm or more in diameter and more than 30 μm in depth in at least 2 consecutive B-scans. Discrepancies were resolved by consensus; if consensus could not be reached, the participant was excluded from the analysis.

### Statistical analysis

Clinical characteristics and examination measurements were compared among groups using analysis of variance (ANOVA) and post-hoc Tukey honestly significant difference tests. Categorical variables were compared using the chi-square test. Univariable logistic regression analysis was performed to identify parameters associated with the Mvd-D. Multivariable logistic regression analysis was performed using covariates with a *P* value of less than 0.10 in the univariable analysis. The kappa (κ) coefficient was calculated to evaluate interobserver agreement in determining the Mvd-D and focal LC defects.[33] All statistical analyses were performed with SPSS Version 24 (IBM Corp., Armonk, NY). *P* values less than 0.05 were considered statistically significant.

## Results

One hundred twenty-three eyes of 123 POAG patients are included in this report. Of the 149 eyes of 149 subjects that initially met patient eligibility criteria, 11 eyes were excluded because of poor-quality OCTA images, 3 eyes because of poor-quality SD-OCT scans, and 12 eyes because of poor-quality SS-OCT scans. One eye was excluded because of failure of the two observers to reach consensus for the determination of the focal LC defect. Inter-observer agreement for determination of Mvd-D, Mvd-P, and focal LC defects was excellent (Mvd-D: (kappa = 0.89; *P* < 0.001; Mvd-P: kappa = 0.88; *P* < 0.001; and focal LC defects: kappa = 0.84; *P* < 0.001).[33]

Forty-four (36%) eyes of 123 eyes of POAG patients were classified as the no Mvd-D group (Figs 1 and 3D), and 42 (34%) and 37 (30%) eyes of the remaining 79 eyes were classified as the Mvd-D group (Fig 3A) and the pseudo-Mvd-D group (Fig 3B and 3C), respectively. In the eyes of pseudo-Mvd-D group, the anterior LC portion was not clearly identified because of shadowing of the neuroretinal rim (26 eyes; Fig 3B) and/or retinal vessels (20 eyes; Fig 3C). The comparisons among these 3 groups are shown in Table 1. Eyes in the Mvd-D group showed significantly lower IOP, worse VF mean deviation (MD), worse PSD, larger C/D ratio, thinner cpRNFL, and lower cpVD than eyes in the pseudo-Mvd-D group or the no Mvd-D group (IOP, *P* = 0.008; other covariates, *P* < 0.001). The vessel densities inside the optic disc and the SSI were significantly lower in the Mvd-D group and the pseudo-Mvd-D group than the no Mvd-D group (optic disc vessel density, *P* < 0.001; SSI, *P* = 0.004). All other covariates were not significantly different between the pseudo-Mvd-D and no Mvd-D group. This implies that the pseudo-Mvd-D and no Mvd-D eyes had similar characteristics in our study.

**Table 1:** Comparison of the demographic and test results between primary open-angle glaucoma patients with and without microvasculature dropout inside the optic disc (Mvd-D).

Variables	A: Mvd-D group (n = 42)	B: Pseudo-Mvd-D group (n = 37)	C: No Mvd-D group (n = 44)	*P* value	Post hoc
Age (years)	72.1±11.5	73.2±12.2	70.2±11.1	0.49	
Gender (male/female)	24/18	20/17	22/22	0.80	
Axial length (mm)	24.33±1.64	23.89±1.33	24.29±1.21	0.33	
CCT (μm)	527.7±45.7	534.3±36.7	537.8±42.4	0.54	
Ethnicity(White/Black/Asian), no.	28/7/7	31/3/3	27/13/4	0.084	
Self-reported history of diabetes,no. (%)	4 (10%)	3 (8%)	7 (16%)	0.49	
Self-reported history of hypertension,no. (%)	17 (40%)	20 (54%)	26 (59%)	0.21	
No. of topical glaucoma medications	2.0±1.3	2.0±1.2	1.7±1.3	0.39	
History of glaucoma surgery, no. (%)	17 (40%)	12 (32%)	13 (30%)	0.55	
IOP (mmHg)	11.9±5	13.6±4.1	14.9±3.8	**0.008**	A<B = C
Systolic BP (mmHg)	126.5±15.9	127±14.7	127±14.7	0.98	
Diastolic BP (mmHg)	76.7±9.3	79.5±10.2	80.6±10.3	0.18	
MOPP (mmHg)	50.3±9.1	49.9±7.8	49.2±7.7	0.82	
Disc hemorrhage, no. (%)	4 (10%)	7 (19%)	10 (23%)	0.25	
Visual field MD (dB)	-11.34±7.93	-5.91±5.46	-4.41±3.43	**<0.001**	A<B = C
Visual field PSD (dB)	8.79±4.04	5.93±3.92	5.3±3.3	**<0.001**	A>B = C
Disc area (mm^2^)	2.03±0.38	1.98±0.49	2.04±0.55	0.82	
Cup-to-disc ratio	0.70±0.11	0.57±0.18	0.55±0.18	**<0.001**	A>B = C
Average cpRNFL thickness (μm)	66.2±11.7	77±11.4	75.8±11.5	**<0.001**	A<B = C
Average cpVD (%)	51.3±6.7	56.5±5.4	57.2±5.3	**<0.001**	A<B = C
Whole-depth vessel density within the optic disc (%)	52.3±6.8	53.5±5.9	58.8±4.1	**<0.001**	A = B<C
Focal lamina cribrosa defect, no. (%)	34 (81%)	15 (41%)	18 (41%)	**<0.001**	
Mvd-P, no. (%)	33 (79%)	7 (19%)	5 (11%)	**<0.001**	
SSI (%)	60.7±8.8	58.9±7.8	65.1±9.0	**0.004**	A = B<C

BP = blood pressure; CCT = central corneal thickness; cpRNFL = circumpapillary retinal nerve fiber layer; cpVD = circumpapillary vessel density; IOP = intraocular pressure; MD = mean deviation; MOPP = mean ocular perfusion pressure; Mvd = microvasculature dropout; Mvd-P = Mvd in the parapapillary deep-layer; PSD = pattern standard deviation; SSI = signal strength index.

Data are mean ± standard deviation, unless otherwise indicated. Values with statistical significance are shown in bold.

The univariable and multivariable logistic regression analyses of the association between the presence of Mvd-D and clinical and ocular characteristics are presented in Table 2. Two types of logistic regression analyses were performed to test factors associated with the presence of Mvd-D. In the first analysis, pseudo-Mvd-D group was included in the no Mvd-D group in Table 2 because the pseudo-Mvd-D and no Mvd-D groups were identical in terms of the absence of Mvd-D. In the second analysis pseudo-Mvd-D group was excluded (S1 Table) to determine whether the results with and without the pseudo-Mvd-D were similar when the Mvd-D and no Mvd-D groups were compared. Based on univariable logistic regression analysis, the presence of Mvd-D was associated significantly with lower IOP (*P* = 0.007; *P* = 0.005), larger C/D ratio (both *P* < 0.001), worse VF MD (both *P* < 0.001), lower cpVD (both *P* < 0.001), thinner cpRNFL (*P* < 0.001; *P* = 0.001), presence of focal LC defect (both *P* < 0.001), and presence of Mvd-P (both *P* < 0.001). Lower SSI was associated significantly with the presence of Mvd-D only when the eyes in the pseudo-Mvd-D group were excluded from the analysis (*P* = 0.026).

**Table 2:** Logistic regression testing factors associated with the presence of microvasculature dropout inside the optic disc in glaucoma eyes (Total 123 eyes of 123 patients).

	Univariable Model		Multivariable Model 1* with Cup-to-disc Ratio Included		Multivariable Model 2* with Visual Field MD Included		Multivariable Model 3* with cpVD Included		Multivariable Model 4* with cpRNFL Thickness Included	
	Odds Ratio (95% CI)	*P*	Odds Ratio (95% CI)	*P*	Odds Ratio (95% CI)	*P*	Odds Ratio (95% CI)	*P*	Odds Ratio (95% CI)	*P*
Age, per 1-yr older	1.01 (0.97, 1.04)	0.78								
White race (vs. nonwhite)	0.79 (0.36, 1.77)	0.57								
Female gender (vs. male)	0.81 (0.38, 1.71)	0.58								
CCT, per 1-μm thicker	1.00 (0.99, 1.00)	0.29								
Axial length, per 1-mm longer	1.12 (0.85, 1.46)	0.43								
IOP, per 1 mmHg lower	**1.14 (1.03, 1.25)**	**0.007**	1.07 (0.95, 1.22)	0.28	1.04 (0.92, 1.17)	0.57	1.04 (0.92, 1.18)	0.50	1.06 (0.95, 1.20)	0.30
Systolic BP, per 1 mmHg higher	1.00 (0.97, 1.02)	0.85								
Diastolic BP, per 1 mmHg lower	1.03 (1.00, 1.08)	0.079	1.06 (1.00, 1.13)	0.060	1.06 (0.99, 1.12)	0.084	1.05 (0.99, 1.11)	0.14	1.05 (0.99, 1.11)	0.11
MOPP, per 1 mmHg higher	1.01 (0.97, 1.06)	0.64								
Diabetes, presence	0.75 (0.22, 2.54)	0.64								
Hypertension, presence	0.52 (0.24, 1.10)	0.088	0.74 (0.25, 2.21)	0.59	0.59 (0.21, 1.63)	0.31	0.51 (0.18, 1.42)	0.20	0.57 (0.21, 1.58)	0.28
Disc hemorrhage, presence	0.40 (0.12, 1.27)	0.12								
Disc area, per 1mm^2^ higher	1.10 (0.50, 2.42)	0.81								
Cup-to-disc ratio, per 1% higher	**1.07 (1.03, 1.11)**	**<0.001**	**1.08 (1.03, 1.13)**	**0.001**	n/a	n/a	n/a	n/a	n/a	n/a
Visual field MD, per 1 dB worse	**1.18 (1.09, 1.27)**	**<0.001**	n/a	n/a	**1.09 (1.00, 1.19)**	**0.048**	n/a	n/a	n/a	n/a
cpVD in RNFL, per 1% lower	**1.18 (1.09, 1.27)**	**<0.001**	n/a	n/a	n/a	n/a	1.07 (0.98, 1.18)	0.12	n/a	n/a
Average cpRNFL thickness, per 1 μm thinner	**1.09 (1.04, 1.12)**	**<0.001**	n/a	n/a	n/a	n/a	n/a	n/a	1.04 (0.99, 1.09)	0.10
SSI, per 1% higher	0.98 (0.94, 1.02)	0.35								
Focal lamina cribrosa defect, presence, presence	**6.18 (2.54, 15.03)**	**<0.001**	**9.05 (2.32, 35.29)**	**0.002**	**5.55 (1.67, 18.40)**	**0.005**	**5.42 (1.68, 17.49)**	**0.005**	**6.01 (1.83, 19.76)**	**0.003**
Parapapillary deep-layer microvasculature dropout	**21.08 (8.08, 54.99)**	**<0.001**	**10.33 (3.45, 30.96)**	**<0.001**	**8.92 (3.14, 25.39)**	**<0.001**	**9.52 (3.35, 27.05)**	**<0.001**	**9.57 (3.40, 26.93)**	**<0.001**

BP = blood pressure; CCT = central corneal thickness; CI = confidence interval; cpRNFL = circumpapillary retinal nerve fiber layer; cpVD = circumpapillary vessel density; IOP = intraocular pressure; MD = mean deviation; MOPP = mean ocular perfusion pressure; SSI = signal strength index.

Values with statistical significance are shown in bold.

*Adjusted for all variables with P < 0.1 in univariate regresion model.

**Fig 4:**
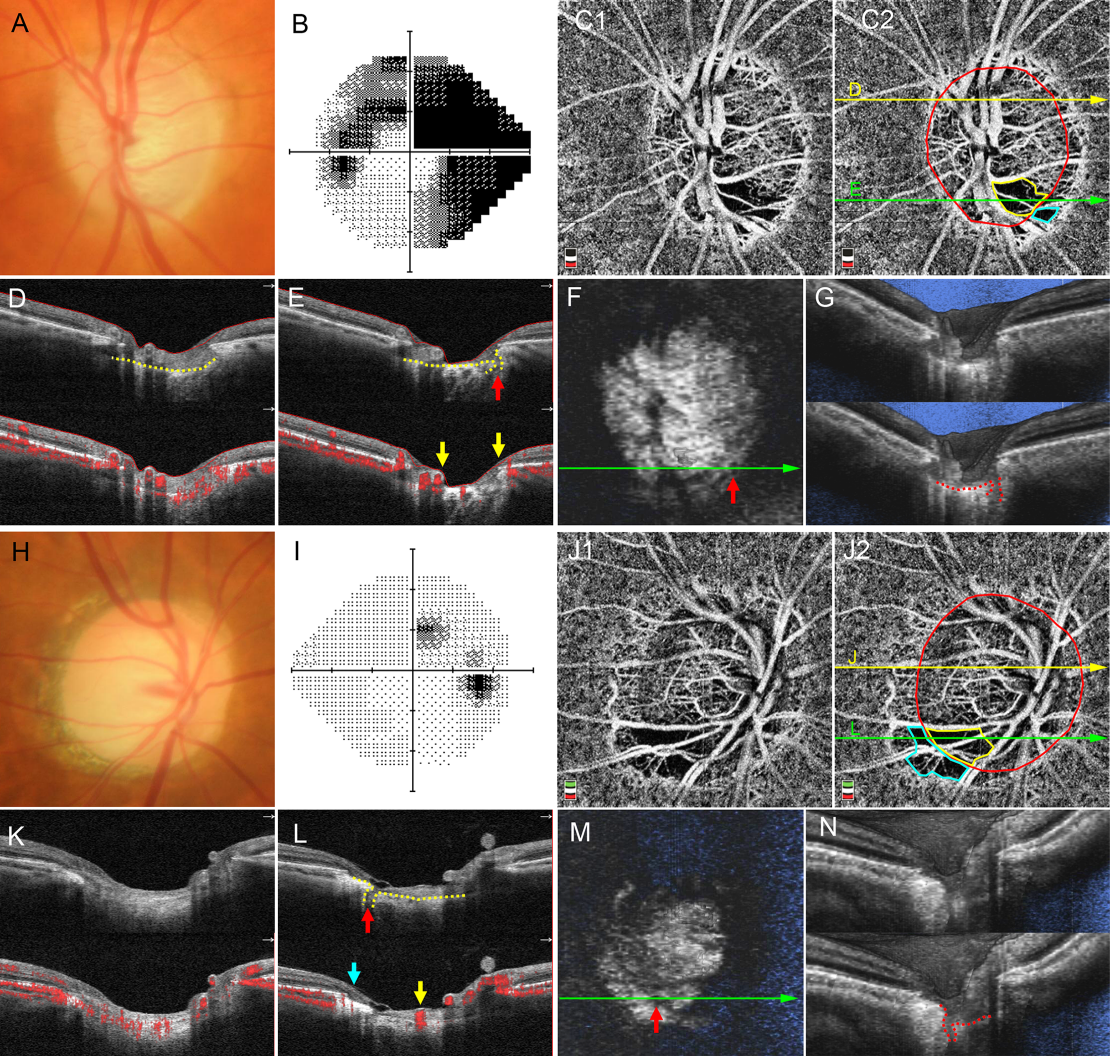
Representative cases of eyes with microvasculature dropout (Mvd) inside the optic disc (Mvd-D). **A―G**, Left eye of a 74-year-old man with primary open-angle glaucoma (POAG). **A**, Neuroretinal rim thinning is observed at the temporal area of the optic disc. **B**, Humphrey Visual Field Analyzer with the 24–2 Swedish interactive threshold algorithm standard (HFA24-2) grayscale image showing severe visual field defects (visual field mean deviation [MD], -16.91 dB). **C**, Optical coherence tomography (OCT) angiography (OCTA) en face projection image with whole-signal-mode. Red ellipse indicates optic disc border. Yellow and aqua borders indicate Mvd-D and Mvd in the parapapillary area (Mvd-P), respectively. **D, E**, B-scan OCT images and cross-sectional angiogram images overlying the B-scan image acquired at the yellow (**D**) and green line (**E**) in **C2**. Microvasculature is observed in the prelaminar tissue and anterior lamina cribrosa (LC) in **D**, while no microvasculature is seen in the Mvd area of **E**. Yellow arrows indicate the borders of Mvd-D. Yellow dotted lines show anterior LC surface. Red arrow indicates focal LC defect. **F, G**, En face (F) and horizontal section of volume scan (G) of swept-source OCT images. Red dotted line indicates anterior LC surface, and red arrow shows focal LC defect. **H―N**, Right eye of a 62-year-old woman with POAG. **H**, Inferotemporal rim thinning is observed. **I**, HFA24-2 grayscale image showing mild visual field defects (visual field MD, -1.72 dB). **J**, OCTA en face projection image with whole-signal-mode. Red ellipse indicates optic disc border. Yellow and aqua borders indicate Mvd-D and Mvd-P, respectively. **K, L**, B-scan OCT images and cross-sectional angiogram images overlying the B-scan image acquired at the yellow (**K**) and green line (**L**) in **J2**. Microvasculature is observed in prelaminar tissue and anterior LC in **K**, while no microvasculature is seen in the Mvd area of **L**. Yellow and aqua arrows indicate the borders of Mvd-D and Mvd-P, respectively. Yellow dotted lines show anterior LC surface, and red arrow indicates focal LC defect. **M, N**, En face (M) and horizontal section of volume scan (N) of swept-source OCT images. Red dotted line indicates anterior LC surface, and red arrow shows focal LC defect.

C/D ratio, VF MD, cpVD, and cpRNFL thickness were significantly correlated (*P* < 0.001, respectively); therefore, these 4 variables were included separately in the multivariable model to avoid multicollinearity (Table 2 and S1 Table). Regardless of which of the 4 variables were included in the model, in the multivariable analysis using a total of 123 eyes, the presence of a focal LC defect and the presence of Mvd-P were associated with the presence of Mvd-D, with odds ratio (OR) ranging from 5.4 to 9.1 and 8.9 to 10.3, respectively (Fig 4). Larger C/D ratio (OR, 1.08; *P* = 0.001) and worse VF MD (OR, 1.09; *P* = 0.048) also remained as significant factors associated with the presence of Mvd-D.

Similarly, in the multivariable analysis excluding the eyes of pseudo-Mvd-D group, in each of the 4 multivariable models, the presence of a focal LC defect and Mvd-P remained as significant factors associated with the presence of Mvd-D. Larger C/D ratio and lower SSI were also associated significantly in some analyses, but the other factors were not (S1 Table).

The topographic characteristics of the focal LC defect and Mvd-P in eyes with Mvd-D are shown in Table 3. In 31 eyes with superior or inferior focal Mvd-D, 25 eyes (81%) and 26 eyes (84%) showed focal LC defects and Mvd-P in the same quadrant, respectively (Fig 4C–4G and 4J–4N).

**Table 3:** Topographic characteristics of lamina cribrosa defect and parapapillary microvasculature dropout (Mvd-P) in eyes with microvasculature dropout inside the optic disc (Mvd-D).

	Location of the Mvd-D		
	Only IT (n = 24)	Only ST (n = 7)	IT and ST (n = 11)
Focal LC defect			
Only IT, no.	13	1	3
Only ST, no.	0	1	0
IT and ST, no.	8	3	5
None, no.	3	2	3
Mvd-P			
Only IT, no.	22	0	1
Only ST, no.	0	3	0
IT and ST, no.	0	1	6
None, no.	2	3	4

IT = inferotemporal; LC = lamina cribrosa; ST = superotemporal.

## Discussion

The results of this study demonstrate that Mvd-D was observed more frequently in POAG eyes with more severe glaucomatous damage than less damaged eyes. Further, Mvd-D was independently associated with both focal LC defect and Mvd-P, even after controlling for severity of disease. These findings suggest that there might be close relationships among Mvd-D, Mvd-P, and focal LC defects.

There have been several reports investigating the microvasculature within the glaucomatous optic disc using OCTA, and it has been reported that the vessel density and flow index in the prelaminar tissue and the whole depth below the ILM are reduced in these eyes.[10,12,26,34,35] However, current OCTA technology has some possible limitations, especially in evaluation of the optic disc. First, there are projection artifacts in which the superficial blood vessels project to deeper tissue. Recently, a post-processing algorithm has been proposed to reduce projection artifacts.[36,37] However, it has not been clarified how this algorithm improves OCTA images of deeper tissue (e.g. LC) within the optic disc. In the current study, we focused on and evaluated qualitatively the complete loss of microvasculature within the optic disc of glaucoma eyes. The whole-signal-mode was used to evaluate microvasculature to exclude the influence of projection artifacts, although there is a possibility that microvasculature in the superficial layer (i.e. neuretinal rim) can mask the Mvd in the deep layer (e.g. LC) in whole-signal-mode imaging. Second, there is shadowing by vessels or neuroretinal rim. OCTA signals are produced from variation in OCT scans; therefore, the absence of OCTA signals in areas without OCT signals does not mean that microvasculature is absent. The absence of OCTA signals in an area with OCT signals has two possible explanations: 1) no microvasculature, or 2) undetectably small OCTA signals. As shown in Figs 3 and 4, some areas showed microvasculature inside the LC, but others did not. In consideration of these results, the OCTA hypo-density area was separately analyzed (Mvd-D and pseudo-Mvd-D), based on the visibility of the anterior portion of LC, to remove the possible effects of shadowing to the greatest possible extent. Because the whole-signal-mode OCTA images at the optic disc potentially contain the components of prelaminar tissue and LC itself, the microvascular reduction in the current study may reflect not only the prelaminar microvasculature but also the microvascular component at the anterior portion of the LC. Nonetheless, it should be noted that we cannot determine whether there is any microvasculature present or whether there is undetectable microvasculature less than threshold in the area with complete loss of OCTA microvasculature.

Similar findings as the Mvd-D and Mvd-P assessed by OCTA have been described in previous reports using fluorescein angiography or indocyanine green angiography.[4–8,38] With fluorescein angiography, three types of defects have been described: local filling defects, slow filling, and increased leakage.[4–8] The Mvd assessed in the current study corresponds to the filling defects, and delayed filling and leakage could not be evaluated with current OCTA technology. Recently, Lee et al. reported that the Mvd-P in OCTA was almost identical to the peripapillary filling defects observed with indocyanine green angiography.[39] In the images of their study, these filling defects inside the optic disc detected with indocyanine green angiography were similar to those visualized with OCTA. Although filling defects in fluorescein or indocyanine green angiography are known to be relatively specific for glaucoma, the association between the filling defects in the optic disc and LC defects has not been investigated, because detection of focal LC defects has only recently become possible using enhanced-depth imaging (EDI)-OCT or SS-OCT.[16–22]

We found that the Mvd-D was related significantly and topographically to focal LC defects and Mvd-P. Focal LC defects have been reported to be more likely to be found in eyes with normal tension glaucoma,[19] a past history of disc hemorrhage,[16,19] worse VF damage,[19] and high myopia.[16,21] The development of focal LC defects may cause the mechanical disruption of the capillaries inside the LC and affect the microvasculature architecture.[15,22] In 79% of the eyes with Mvd-D, Mvd-P was observed in the same quadrant as Mvd-D (Table 3). The parapapillary area, prelaminar tissue, and LC are perfused by short posterior ciliary arteries.[40–43] These facts suggest that these two locations of microvascular defects are closely related in their development. However, our results could not clarify whether the focal LC defect is a cause or a result of Mvd-D and Mvd-P. Prospective longitudinal studies should clarify the temporal association between focal LC defect and Mvd-D and reveal whether vascular dysfunction is a risk factor for the development/progression of glaucoma.

In the current study, IOP was significantly lower in the Mvd-D group than the other groups (Table 1). One possible explanation for the difference in IOP is that the Mvd-D group was treated more aggressively. It was reasonable that the rate of previous glaucoma surgery and topical glaucoma medications were higher in the Mvd-D, because the eyes in the Mvd-D group had significantly more severe VF defects than the other groups (Table 1, *P* < 0.001). Further investigations are needed to clarify this issue.

The current study has several limitations. First, the loss of microvasculature was investigated using whole-signal-mode images. Consequently, only eyes with unambiguous Mvd, to reduce the potential influence of the artifacts with vascular/rim shadowing or projection onto the underlying tissues, were identified.[26] This method ensures that the microvasculature in the anterior portion of the LC is definitely lost in the Mvd area. In contrast, it is difficult to detect Mvd in the LC below the area where there is existing prelaminar microvasculature. Further, because only the eyes with well-visualized LC in SS-OCT were included, eyes with large discs may have been preferentially selected; this might mask the association between disc size and Mvd-D as a selection bias. A previous report using the microsphere technique in monkey eyes showed that blood flow in the LC was nearly equivalent to that in prelaminar tissue.[44] Further studies using improved OCTA systems may help to reveal the precise relationship between blood flow in prelaminar tissue and LC. Second, the OCTA system in the current study was based on SD-OCT. Although a previous study showed that even normal SD-OCT, not EDI-OCT or SS-OCT, can visualize the full thickness of the LC in approximately 60% of the eyes, its depth penetration is limited for LC visualization because of absorption and scattering with a probe light operated at approximately 800 nm.[45] OCTA with high penetration imaging, such as EDI-OCT or SS-OCT, might be more appropriate for that purpose.[16,21,46–48] Third, qualitative assessment for Mvd was performed only in the temporal side of the optic disc. It should be noted that deeper structures of the nasal optic disc are difficult to visualize by SD-OCT due to densely packed large retinal vessels and thick rim.[29,30] Further, the temporal optic disc, especially the inferotemporal and superotemporal quadrants, tends to be more affected in glaucoma. For this reason, the temporal optic disc was selected for evaluation. However, it is possible that some Mvds in the nasal optic disc with this approach were missed. It was important to use visualization of the anterior LC portion in OCT B-scan images in the classification of Mvd-D groups. This is because OCTA microvasculature loss in the eyes without clear OCT visualization might only depend on the quality of the scanning images. In fact, the pseudo-Mvd-D eyes had similar characteristics to the no Mvd-D eyes, except for optic disc vessel density and SSI; both of these are associated with imaging performance. However, classification using OCT visualization potentially means that focal LC defects are more easily detectable in Mvd-D eyes than in pseudo-Mvd-D eyes and no Mvd-D eyes even though focal LC defects were evaluated using SS-OCT for its high-penetration imaging.

In conclusion, OCTA-derived Mvd-D was strongly and topographically associated with the presences of Mvd-P and focal LC defects. Mvd-D and Mvd-P may be developmentally related to focal LC damage in glaucoma.

## Supporting information

S1 Table Logistic regression testing factors associated with the presence of microvasculature dropout inside the optic disc (Mvd-D) in glaucoma eyes (86 eyes of 86 patients in Mvd-D group and no Mvd-D group). (DOCX)

S1 Dataset The raw data of all subjects. (XLSX)
