# Supplementary material for: Optic disc microvasculature dropout in primary open-angle glaucoma measured with optical coherence tomography angiography
Source: PLoS One. 2018 Aug 7;13(8):e0201729. doi: 10.1371/journal.pone.0201729 (PMC6080778; doi:10.1371/journal.pone.0201729)
Supplement: S1 Table — (DOCX) [file pone.0201729.s001.docx]

**S1 Table.** Logistic Regression Testing Factors Associated with the Presence of Microvasculature Dropout inside the Optic Disc (Mvd-D) in Glaucoma Eyes (86 Eyes of 86 Patients in Mvd-D group and no Mvd-D group)

|  | Univariable Model | | Multivariable Model 1*  with Cup-to-disc Ratio Included | | Multivariable Model 2*  with Visual Field MD Included | | Multivariable Model 3*  with cpVD Included | | Multivariable Model 4*  with cpRNFL Thickness Included | |
| --- | --- | --- | --- | --- | --- | --- | --- | --- | --- | --- |
|  | Odds Ratio (95% CI) | *P* | Odds Ratio (95% CI) | *P* | Odds Ratio (95% CI) | *P* | Odds Ratio (95% CI) | *P* | Odds Ratio (95% CI) | *P* |
| Age, per 1-yr older | 1.02 (0.98, 1.06) | 0.41 |  |  |  |  |  |  |  |  |
| White race (vs. nonwhite) | 1.26 (0.52, 3.05) | 0.61 |  |  |  |  |  |  |  |  |
| Female gender (vs. male) | 0.75 (0.32, 1.76) | 0.51 |  |  |  |  |  |  |  |  |
| CCT, per 1-μm thicker | 1.00 (0.99, 1.01) | 0.29 |  |  |  |  |  |  |  |  |
| Axial length, per 1-mm longer | 1.02 (0.76, 1.37) | 0.91 |  |  |  |  |  |  |  |  |
| IOP, per 1 mmHg lower | **1.16 (1.30, 1.04)** | **0.005** | 1.21 (1.48, 0.98) | 0.071 | 1.17 (1.42, 0.96) | 0.13 | 1.18 (1.42, 0.98) | 0.088 | 1.19 (1.45, 0.98) | 0.080 |
| Systolic BP, per 1 mmHg higher | 1.00 (0.97, 1.03) | 0.87 |  |  |  |  |  |  |  |  |
| Diastolic BP, per 1 mmHg lower | 1.04 (1.09, 1.00) | 0.075 | 1.09 (1.2, 0.99) | 0.088 | 1.06 (1.15, 0.98) | 0.14 | 1.06 (1.14, 0.98) | 0.16 | 1.07 (1.16, 0.98) | 0.12 |
| MOPP, per 1 mmHg higher | 1.02 (0.97, 1.07) | 0.55 |  |  |  |  |  |  |  |  |
| Diabetes, presence | 0.56 (0.15, 2.06) | 0.38 |  |  |  |  |  |  |  |  |
| Hypertension, presence | 0.47 (0.20, 1.11) | 0.086 | 0.3 (0.06, 1.45) | 0.13 | 0.4 (0.1, 1.65) | 0.21 | 0.33 (0.08, 1.31) | 0.11 | 0.38 (0.09, 1.57) | 0.18 |
| Disc hemorrhage, presence | 0.36 (0.10, 1.25) | 0.11 |  |  |  |  |  |  |  |  |
| Disc area, per 1mm^2^ higher | 0.97 (0.39, 2.40) | 0.94 |  |  |  |  |  |  |  |  |
| Cup-to-disc ratio, per 1% higher | **1.08 (1.04, 1.12)** | **<0.001** | **1.11 (1.03, 1.19)** | **0.004** | n/a | n/a | n/a | n/a | n/a | n/a |
| Visual field MD, per 1 dB worse | **1.25 (1.41, 1.12)** | **<0.001** | n/a | n/a | 1.11 (1.29, 0.95) | 0.19 | n/a | n/a | n/a | n/a |
| cpVD in RNFL, per 1% lower | **1.18 (1.28, 1.09)** | **<0.001** | n/a | n/a | n/a | n/a | 1.04 (1.19, 0.9) | 0.62 | n/a | n/a |
| Average cpRNFL thickness, per 1 μm thinner | **1.08 (1.12, 1.03)** | **0.001** | n/a | n/a | n/a | n/a | n/a | n/a | 1.06 (1.13, 0.99) | 0.11 |
| SSI, per 1% lower | **1.05 (1.11, 1.01)** | **0.026** | **1.12 (1.24, 1.01)** | **0.027** | 1.09 (1.2, 0.99) | 0.067 | **1.11 (1.22, 1.01)** | **0.039** | **1.11 (1.22, 1.01)** | **0.031** |
| Focal lamina cribrosa defect, presence, presence | **6.14 (2.31, 16.31)** | **<0.001** | **9.65 (1.53, 60.98)** | **0.016** | **5.93 (1.23, 28.65)** | **0.027** | **6.25 (1.34, 29.16)** | **0.020** | **7.64 (1.47, 39.78)** | **0.016** |
| Parapapillary deep-layer microvasculature dropout | **28.60 (8.72, 93.77)** | **<0.001** | **27.85 (4.81, 161.07)** | **<0.001** | **14.83 (2.93, 75.02)** | **0.001** | **20.41 (3.85, 108.28)** | **<0.001** | **17.09 (3.72, 78.54)** | **<0.001** |

BP= blood pressure; CCT = central corneal thickness; CI = confidence interval; cpRNFL = circumpapillary retinal nerve fiber layer; cpVD = circumpapillary vessel density; IOP = intraocular pressure; MD = mean deviation; MOPP = mean ocular perfusion pressure; SSI = signal strength index.

Values with statistical significance are shown in bold.

*Adjusted for all variables with P < 0.1 in univariate regresion model.
